# The enteric bacterial metabolite propionic acid alters brain and plasma phospholipid molecular species: further development of a rodent model of autism spectrum disorders

**DOI:** 10.1186/1742-2094-9-153

**Published:** 2012-07-02

**Authors:** Raymond H Thomas, Melissa M Meeking, Jennifer R Mepham, Lisa Tichenoff, Fred Possmayer, Suya Liu, Derrick F MacFabe

**Affiliations:** 1 The Kilee Patchell-Evans Autism Research Group, Department of Psychology, University of Western Ontario, London, ON, N6A 5C2, Canada; 2 Department of Obstetrics/Gynecology and Biochemistry, University of Western Ontario, London Health Sciences Center, London, ON, Canada; 3 Biological Mass Spectrometry Laboratory, Department of Biochemistry, University of Western Ontario, London, ON, Canada

**Keywords:** Locomotor activity, Membrane fluidity, Gap junction, Plasmalogens, Docosahexaenoic acid, Oxidative stress

## Abstract

Gastrointestinal symptoms and altered blood phospholipid profiles have been reported in patients with autism spectrum disorders (ASD). Most of the phospholipid analyses have been conducted on the fatty acid composition of isolated phospholipid classes following hydrolysis. A paucity of information exists on how the intact phospholipid molecular species are altered in ASD. We applied ESI/MS to determine how brain and blood intact phospholipid species were altered during the induction of ASD-like behaviors in rats following intraventricular infusions with the enteric bacterial metabolite propionic acid. Animals were infused daily for 8 days, locomotor activity assessed, and animals killed during the induced behaviors. Propionic acid infusions increased locomotor activity. Lipid analysis revealed treatment altered 21 brain and 30 blood phospholipid molecular species. Notable alterations were observed in the composition of brain SM, diacyl mono and polyunsaturated PC, PI, PS, PE, and plasmalogen PC and PE molecular species. These alterations suggest that the propionic acid rat model is a useful tool to study aberrations in lipid metabolism known to affect membrane fluidity, peroxisomal function, gap junction coupling capacity, signaling, and neuroinflammation, all of which may be associated with the pathogenesis of ASD.

## Introduction

Autism spectrum disorders (ASD) are a family of disorders characterized by stereotypic and restrictive patterns of behavior, deficits in social interactions, and impairments in language development and communication skills [1]. Several recent studies have indicated that interactions between genetic, metabolic, immunological, gastrointestinal, environmental, and behavioral factors may be associated with the pathogenesis of ASD [2-6]. Our research group has been developing a novel rodent model of autism to examine enteric bacterial metabolites as possible environmental triggers in ASD. Such a model could prove useful in investigating how the interactions between the above factors may contribute to the pathogenesis of ASD [7-12], and identify potential biomarkers that could be used in early detection and screening for this disorder.

The central premise of this model is that propionic acid (PPA) and/or related enteric fatty acids may be candidate environmental factors involved in the development of some types of ASD. Propionate and other short chain fatty acids (for example butyrate, acetate) are produced in the body during normal cellular metabolism and following enteric bacterial fermentation of dietary carbohydrates and proteins [13]. PPA producing enteric bacteria, including unique *ClostridialDesulfovibrio*, and *Bacteriodetes* species, have been isolated from patients with regressive ASD [2,14]. Propionate is also present naturally in a variety of foods and is a common food preservative in refined wheat and dairy products [15]. Under normal circumstances these short chain fatty acids are primarily metabolized in the liver. However, if there are genetic and/or acquired aberrations in metabolism [7,16], higher than normal levels of short chain fatty acids can be present in the circulating blood, and can cross the gut-blood and blood brain barriers passively and/or actively via high affinity transporters [17]. Under these conditions, short chain fatty acids can concentrate intracellularly, particularly in acidotic conditions [18,19], where they may have deleterious effects on brain development and function [13,20,21]. This could be important in the context of ASD, since PPA is known to affect cell signaling [22], neurotransmitter synthesis and release [20], mitochondrial function/CoA sequestration [16], lipid metabolism [23] immune function [24], gap junction modulation [19], and gene expression [25], all of which have been implicated in ASD [7,25-28].

Previous results from our work developing this model indicate that brief, intracerebroventricular infusions with PPA, but not control compounds (that is, propanol) produced short bouts of behavioral (hyperactivity, perserveration, object fixation, social impairments) and electrophysiological (seizure, caudate spiking) effects, coupled with biochemical (increased oxidative stress, reduced glutathione) and neuropathological (innate neuroinflammation) alterations in adult rats, consistent with those seen in ASD [7-11]. Recent findings with this model indicate infusions with PPA or butyrate altered the brain acylcarnitine and phospholipid profiles [12]. Interestingly, the alterations in rat brain lipids noted in this study showed a number of similarities to those documented in blood from ASD patients [5,29-32].

In the above studies, phospholipid fatty acid composition was determined by analyzing fatty acid acyl groups following hydrolysis of the separated phospholipid classes. Though the standard accepted method of lipid analysis, this method destroys the phospholipid structure, abolishing the ability to establish the individual phospholipid molecular species distribution of the samples. Recently, it has become possible to apply electrospray ionization mass spectrometry to analyze phospholipids as their intact molecular species, allowing for identification of individual and patterns of specific lipids [33-35]. This is potentially important considering there are presently no reliable biomarkers for the detection of ASD, the etiology of ASD is unclear, and altered lipid profiles have been reported both for ASD patients [5,27,29,32,36] and in the PPA rodent model of ASD [12]. Therefore, there is a need to determine: (1) whether or not the intact phospholipid molecular species are altered in relation to behaviors, to further validate this animal model; and (2) how these altered phospholipid profiles may relate to the pathogenesis of ASD.

The development of liquid chromatography electrospray ionization mass spectrometry (LC-ESI/MS) has made it possible to directly analyze phospholipids as intact molecular species, and preserve the information inherent in their chemical structure [33,35]. Herein, we used ESI/MS to determine how the intact blood and brain phospholipid molecular species are altered during the induction of abnormal (ASD-like) behaviors following PPA infusions in rats.

## Materials and methods

### Subjects

Twenty four Long-Evans rats (Charles River Laboratories, Quebec, Canada), weighing 200–225 g (approximately 47–49 days old) at the start of the experiment, were individually housed at 21 ± 1°C in standard acrylic cages (26 × 48 × 21 cm) and exposed to 12:12 h light–dark cycle (lights on 0700 h to 1900 h). Animals were allowed access to food (Prolab rat chow) and tap water *ad libitum*. All procedures were in accordance with guidelines of the Canadian Council on Animal Care and were approved by the University of Western Ontario Animal Use Subcommittee.

### Surgical implantation of intracerebroventricular (ICV) cannula

To induce anesthesia, animals were placed in a sealed Plexiglas box into which 4 % isoflurane and 2 L/min oxygen flow were introduced. The animal was then placed into a Kopf stereotaxic frame equipped with a gas flow nose cover to maintain anesthesia throughout surgery at 2 % isoflurane and 500 mL/min oxygen flow. Under aseptic conditions, the animal received an implantation of a 23-gauge guide cannula with the tip in the right lateral ventricle (AP 1.3 mm, ML 1.8 mm, DV 3.0 mm) [37]. Cannula placements were in accordance with Paxinos and Watson [37] rat brain atlas and our previous experience [7-11]. A removable obturator sealed the guide cannula until an injection was to be made. To facilitate infusion into the lateral ventricle, the tip of a 30-gauge injection cannula protruded 0.5 mm beyond the tip of the guide cannula. Small stainless steel screws were inserted into the skull surrounding the cannula to provide anchors for dental acrylic, which attached the cannula to the skull. Immediately post-surgery, all rats received a subcutaneous injection of analgesic (Ketoprofin, 1 mL/kg). All animals were allowed 14 days recovery before behavioral testing took place.

### Treatment groups and intracerebroventricular infusion procedure

Following recovery, animals were assigned to one of two groups: PPA treatment (4.0 μL of a 0.26 M solution, *n* = 12), or phosphate buffered saline (PBS) control (4.0 μL, *n* = 12). Sodium propionic acid was dissolved in PBS vehicle and buffered to pH 7.5 using concentrated HCl. Each group received intracerebroventricular (ICV) infusions twice daily, separated by 4 h, for 8 consecutive days. Compounds were infused using a 30-gauge injection cannula connected to a Sage syringe pump with sterile PE10 tubing. The tip of the injection cannula protruded 0.5 mm beyond the tip of the guide cannula. Each injection consisted of 4.0 μL of solution delivered over a period of 1 min. The infusion cannula remained in place for an additional minute before being removed.

### Apparatus: automated activity monitors

Locomotor activity was monitored using three Versamax Animal Activity Monitors (AccuScan Model DCM-8, Columbus, OH, USA). Each monitor consisted of a Plexiglas open field chamber (40 × 40 × 30.5 cm), and a Plexiglas lid with air holes. Located on all four sides of the chamber were 16 infrared beam sensors 2.54 cm apart and 4.5 cm from the floor to measure horizontal movements and on two opposite sides were 16 infrared beams located 15 cm above the chamber floor to measure the vertical movements. Light levels at the floor of each open-field were approximately 900 lux. A VersaMax Analyser (Accuscan Model VSA-16, Columbus, OH, USA) processed relayed data from each automated open-field to a computer located in an adjacent room to the testing chambers [7,8].

### Behavioral testing

The methods used for behavioral assessment presented in this study have been previously reported (2011) [7-12]. Briefly, animals were habituated to the automated activity monitors for two 30-min sessions prior to the treatment sessions. Baseline was recorded on the third day to establish normal activity levels for untreated animals. Locomotor assessments were made for 30 min immediately following the second ICV infusion daily for 7 days. Locomotor activity was assessed using horizontal, vertical, and repetitive measures (stereotypy) across infusion days. The horizontal activity measures analyzed were number of horizontal movements (the number of horizontal movements separated by 1 s stop time), horizontal movement time (length of time in seconds an animal was engaged in horizontal movement), and total distance (total horizontal distance (cm) traveled). The vertical activity measure analyzed was number of vertical movements (number of vertical movements separated by 1 s stop time). The repetitive activity measures were: clockwise revolutions (the number of times an animal ran around in a clockwise circle of at least 2 inches in diameter), counterclockwise revolutions (the number of times an animal ran around in a counterclockwise circle of at least 2 inches in diameter), and the number of stereotypic movements (repeated breaking of the same infrared beam separated by 1 s or more). Fifteen minutes after animals received the final ICV infusion on the eighth treatment day, they were decapitated, brains quickly removed, placed on powdered dry ice, then transported from surgery room to the analytical laboratory for lipid analysis. Samples were then stored in a −70°C freezer for 2 days before lipid analysis. Whole blood collected from the neck following decapitation was heparinized and spun at 3000 × g for 5 min, and the supernatant (plasma) collected for lipid analysis.

### Lipid extraction

Brain tissue (100 mg) ipsilateral to ICV infusion site, containing frontal cortex, striatum, thalamus, and dorsal hippocampus, 1.3 mm posterior to bregma, [37] was ground to fine powder while frozen in liquid nitrogen. The ground brain samples (100 mg) and plasma (100 uL) were transferred to centrifuge tubes containing a mixture (2.5 mL) of chloroform/methanol/0.01 % butylated hydroxytoluene (2:1:0.0003; v/v/wt) and 100 μL of 1 mg/mL 1,2-dipalmitoyl-*sn*-glycero-3-phospho-N,N-dimethylethanolamine (Sigma, St Louis, MO, USA) added as internal standard. The sample and solvent mixture was centrifuged at 10,000 × g for 15 min. Following centrifugation, the supernatant was collected and 1 mL of 0.25 % potassium chloride (KCl) was added. The sample was then incubated in a water bath at 70°C for 10 min. After incubation, the sample was removed, cooled and the aqueous layer (top) was removed with a Pasteur pipette. The remaining layer (organic) containing the lipids was transferred to pre-weighed 4 mL sample vial having a poly-teflon resin lined cap (VWR). The sample was dried under a gentle stream of nitrogen and the vial re-weighed to determine the amount of sample recovered [38,39]. The recovered sample was then resuspended in 1 mL chloroform:methanol (2:1 v/v) and stored at −20°C for analysis (ESI/MS).

### Electrospray ionization mass spectrometry analysis (ESI-MS)

Aliquots of blood and brain samples were analyzed by infusion on a triple quadruple instrument (Micromass QuattroMicro; Waters, Milford. MA, USA) equipped with a Z-spray source. The mass spectrometer was operated in both the positive and negative ion modes, and precursor ion or neutral loss scans specific to the phospholipid classes were used to determine the composition of the molecular species of each class [33,40]. Precursor ion scan (negative mode) of *m/z* 153 was used to detect all glycerophospholipid molecular species, *m/z* 241 used to detect PI molecular species (negative mode), precursor ion scan of *m/z* 196 (negative mode) or neutral loss scan of 141 D (positive mode) used to detect PE molecular species, and neutral loss scan of 87 D (negative mode) or 185 D (positive mode) used to detect PS molecular species; while precursor ion scan of *m/z* 184 in the positive mode was used to detect SM and PC molecular species. The fatty acid composition of the identified molecular species of each phospholipid classes was determined via MS/MS analysis and reference to the literature (LIPID MAPS). The investigated mass range was from 100–1000  *m/z*. The cone voltage was set at 30 V, the collision energy at 30–50 V, source temperature at 80°C and the collision gas at 2xe-3 Mbar. Calibration was performed with sodium iodide and mass error was less than 0.05 Dalton (Da). Flow injection was carried out with chloroform:methanol:water:ammonia (20:70:8:2 v/v/v/v) at a flow rate of 50 uL/min with 20 uL of the sample injected for analysis. Quantitative analysis was conducted using MassLynx 4.0 software (Micromass) and Microsoft Excel by using the signal intensity of the reference standard to normalize the intensities of the identified molecular phospholipid species after correction for ^13^ C isotope effects. Representative external standards for each phospholipid classes (Avanti Polar Lipids, Alabaster, AL, USA) were used to determine the relative amounts of the molecular species within a phospholipid class. The amounts of each molecular species within a phospholipid class are expressed as nanomole percent composition.

### Statistical analysis

A total of 24 animals were used for this study (12 PBS and 12 PPA).

General analysis of variance (ANOVA) was used to determine the effects of treatments on behavior and phospholipid components. Where treatment effects were significant, the means were compared with Fisher’s LSD test, α = 0.05. A one-way ANOVA was conducted on the baseline data to ensure that there were no individual differences in behavior prior to treatment days. The data were analyzed using the Statistix software package (Analytical Software, Tallahassee, FL, USA).

## Results

### Behavioral assessment

**Figure 1:**
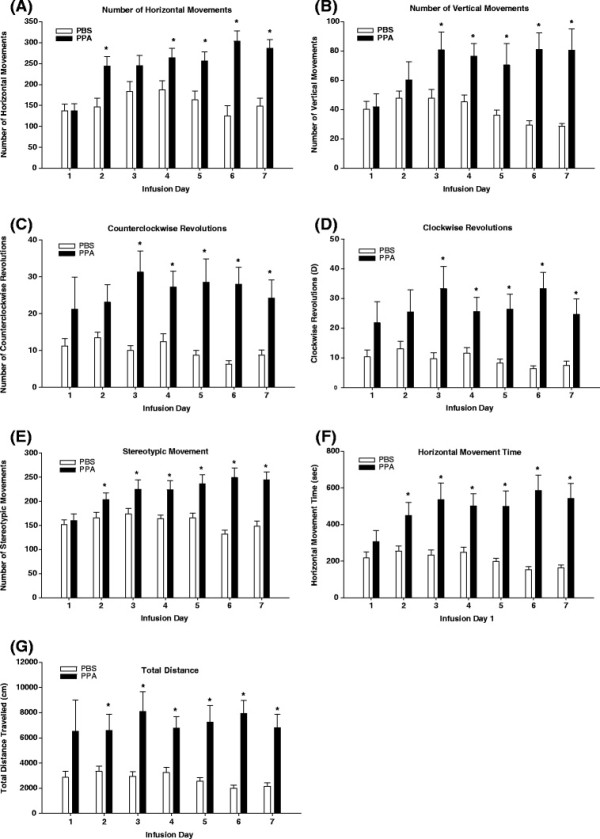
**Number of horizontal movements (A), number of vertical movements (B), counterclockwise revolutions (C), clockwise revolutions (D), number of stereotypic movements (E), horizontal movement time (F), and total distance travelled (G), representing abnormal animal behavior following ICV infusions with PPA and PBS.** Animals received ICV infusions of PPA ( *n* = 12) and PBS ( *n* = 12) twice a day for 7 consecutive days. Behavioral measurements were monitored immediately after each infusion over 30 min. Values represent means ± SE over 7 days. Bars accompanied by asterisks indicate significant difference between treatments at LSD = 0.05, *n* = 12 per treatment group. PBS, phosphate buffered saline solution; PPA, propionic acid.

Locomotor behavior was assessed daily for 7 days, immediately following the second infusion of the day. Prior to behavioral assessment, there was no significant difference in baseline behavior between animals assigned to the PPA and PBS treatment groups. Locomotor activity expressed by horizontal, vertical, and repetitive measures across infusion days, was used to assess animal behavior. Analysis of horizontal activity measures revealed a significant effect of PPA treatment across infusion days for number of horizontal movements, horizontal movement time, and total distance traveled (Figure 1). Propionic acid treated animals traveled further, made more horizontal movements, and spent more time traveling horizontally than PBS treated animals. The number of horizontal movements was significantly higher (*F* (6, 126) = 3.69, *P* <0.001) in rats infused with PPA on infusion days 2, 4, 5, 6, and 7 compared to PBS controls (Figure 1a). A similar pattern was observed for horizontal movement time (*F* (6, 126) = 2.82, *P* <0.001) (Figure 1f) and total distance traveled (*F* (6, 126) = 2.08, *P* <0.001) (Figure 1 g), whereby these behavioral activities were significantly higher in PPA treated animals compared to PBS controls on infusion days 2, 3, 4, 5, 6, and 7. Analysis of the number of vertical movements revealed a significant (*F* (6, 126) = 2.82, *P* <0.001) main effect of PPA treatment across infusion days, indicating PPA treated animals made more vertical movements than PBS treated animals on infusion days 3 through 7 (Figure 1b). The results of the repetitive activity measures showed significant increases in counterclockwise revolutions (*F* (6, 126) = 2.46, *P* <0.001), clockwise revolutions ( *F* (6, 126) = 2.40, *P* <0.001) and number of stereotypic movements ( *F* (6, 126) = 4.05, *P* <0.001) in PPA-treated animals across infusion days. On infusion days 3, 4, 5, 6, and 7, animals infused with PPA made more counterclockwise (Figure 1c) and clockwise (Figure 1d) revolutions than PBS controls. Stereotypy showed a similar trend whereby PPA-treated animals displayed more stereotypic movements than PBS animals on infusions days 2 through 7 (Figure 1e).

### Lipid analysis

**Figure 2:**
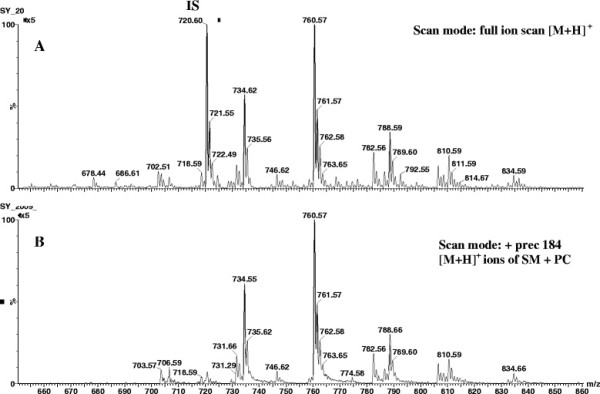
**Positive ion mass spectra of brain lipid extract.** ( **A**) Full ion scan of all lipids present in positive ion mode. ( **B**) Detection of [M + H]^+^ ions of PC and SM molecular species by precursor scanning of *m/z* 184. IS, internal standard; PC, phosphatidylcholine; SM, sphingomyelin.

**Figure 3:**
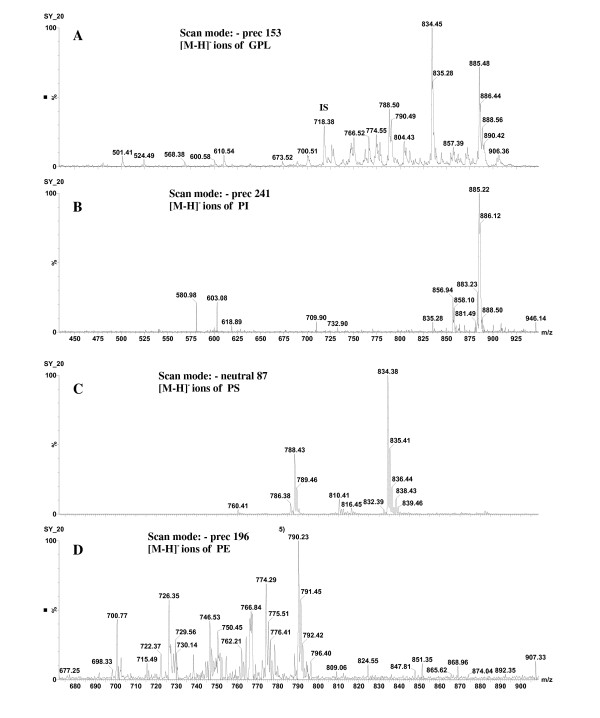
**Negative ion mass spectra of brain lipid extract.** ( **A**) Detection of all [M-H] ^-^ ions of glycerophospholipids by precursor ion scan of *m/z 153*. ( **B**) Detection of PI molecular species by precursor ion scanning of *m/z 241*. ( **C**) Detection of PS molecular species by neutrol loss of 87 D. ( **D**) Detection of PE molecular species by precursor ion scanning of *m/z 196*. GPL, glycerophospholipids; IS, internal standard; PI, phosphatidylinositol; PS, phosphatidylserine; PE, phosphatidylethanolamine.

Behavioral assessment indicated animals developed abnormal (ASD-like) behaviors following PPA infusion (Figure 1). Animals were killed during the expression of these abnormal behaviors on day 8 and the molecular species of five phospholipid classes (SM, PC, PI, PS, and PE) were evaluated to determine whether they were altered during the expression of these behaviors. Phosphatidylcholine and SM molecular species were detected in the positive ion mode (Figure 2), while PI, PS, and PE molecular species were detected in the negative ion mode (Figure 3). Although the same molecular species for each phospholipid class were detected in control and PPA-infused animals, the analyses revealed a quantitative change in these lipid constituents following PPA infusions (Table 1, 2, 3, 4, 5, 6, 7, 8, 9, and 10).

**Table 1:** Changes (percent composition) in rat brain sphingomyelin molecular species following intraventricular infusion with propionic acid (PPA) and phosphate buffered saline (PBS)

**Molecular weight**	**Molecular species**	**Base/acyl species**	**PBS**	**PPA**
703	34:1	18:1/16:0	0.07 ± 0.03	0.10 ± 0.05
733	36:0	18:0/18:0	21.96 ± 0.35	39.13 ± 1.35*
731	36:1	18:1/18:0	6.21 ± 0.67	4.53 ± 1.28
761	38:0	18:0/20:0	38.09 ± 0.84	29.37 ±1.92*
759	38:1	18:1/20:0	16.08 ± 0.19	12.31 ± 3.64*
789	40:0	18:0/22:0	13.03 ± 0.24	9.92 ± 1.31*
811	42:3	18:1/24:2	4.56 ± 0.12	4.64 ± 1.54
**Total%**			**100**	**100**
**∑ Saturates**			**73.07 ± 0.15**	78.41 ± 1.44*
**∑ Monounsat**			**22.37 ± 0.66**	16.94 ± 2.91*
**∑ Polyunsat**			**4.55 ± 0.12**	**4.63 ± 1.54**

Values (nanomole percent by weight composition) represent means ± standard errors. Means in the same row accompanied by asterisks are significantly different between treatments at LSD = 0.05, *n* = 12 per treatment group. Monounsat, monounsaturated fatty acids; PBS, phosphate buffered saline solution; PPA, propionic acid; polyunsat, polyunsaturated fatty acids. Sphingomyelin molecular species were identified using a precursor ion scan of *m/z* 184 in ESI positive mode. The lipid components in the table are arranged based on the molecular species composition.

**Table 2:** Changes (percent composition) in rat plasma sphingomyelin molecular species following intraventricular infusion with propionic acid (PPA) and phosphate buffered saline (PBS)

**Molecular weight**	**Molecular species**	**Base/acyl species**	**PBS**	**PPA**
703	34:1	18:1/16:0	14.05 ± 1.42	4.63 ± 1.10*
733	36:0	18:0/18:0	2.63 ± 0.47	3.40 ± 1.23
731	36:1	18:1/18:0	1.54 ± 0.32	1.65 ± 0.42
761	38:0	18:0/20:0	12.91 ± 0.37	15.92 ± 0.65*
759	38:1	18:1/20:0	8.36 ± 0.56	5.77 ± 0.24*
789	40:0	18:0/22:0	9.44 ± 2.89	10.39 ± 2.03
811	42:3	18:1/24:2	51.07 ± 0.62	58.24 ± 0.68*
**Total%**			**100**	**100**
**∑ Saturates**			**24.98 ± 1.33**	29.72 ± 1.49*
**∑ Monounsat**			**23.95 ± 1.53**	12.05 ± 1.57*
**∑ Polyunsat**			**51.06 ± 0.63**	58.22 ± 0.68*

Values (nanomole percent by weight composition) represent means ± standard errors. Means in the same row accompanied by asterisks are significantly different between treatments at LSD = 0.05, *n* = 12 per treatment group. Monounsat, monounsaturated fatty acids; PBS, phosphate buffered saline solution; PPA, propionic acid; polyunsat, polyunsaturated fatty acids. Sphingomyelin molecular species were identified using a precursor ion scan of *m/z* 184 in ESI positive mode. The lipid components in the table are arranged based on the molecular species composition.

**Table 3:** Changes (percent composition) in rat brain phosphatidylcholine molecular species following intraventricular infusion with propionic acid (PPA) and phosphate buffered saline (PBS)

**Molecular weight**	**Molecular species**	**Plasmalogen or diacyl species**	**PBS**	**PPA**
706	D30:0	16:0/14:0	0.13 ± 0.055	0.13 ± 0.04
734	D32:0	16:0/16:0	19.23 ± 0.34	19.33 ± 0.69
718	P32:1	p16:0/16:1	0.05 ± 0.01	0.06 ± 0.01
762	D34:0	16:0/18:0	18.66 ± 0.49	19.14 ± 0.63
760	D34:1	16:0/18:1	32.80 ± 0.15	33.68 ± 0.16*
746	P34:1	p16:0/18:1	1.54 ± 0.26	1.40 ± 0.32
758	D34:2	16:0/18:2	0.27 ± 0.04	0.69 ± 0.19*
788	D36:1	18:0/18:1	10.53 ± 0.18	9.20 ± 0.15*
774	P36:1	p18:0/18:1	0.50 ± 0.11	0.40 ± 0.11
782	D36:4	16:0/20:4	3.96 ± 0.51	4.81 ± 0.53*
814	D38:2	18:1/20:1	1.21 ± 0.25	1.29 ± 0.11
812	D38:3	18:0/20:3	1.85 ± 0.28	1.74 ± 0.14
810	D38:4	18:0/20:4	4.91 ± 0.26	4.33 ± 0.34
806	D38:6	16:0/22:6	2.98 ± 0.12	2.74 ± 0.10
834	D40:6	18:0/22:6	1.36 ± 0.11	1.06 ± 0.14
**Total%**			**100**	**100**
**∑ Saturates**			**18.78 ± 0.48**	**19.27 ± 0.60**
**∑ Monounsat**			**62.56 ± 0.51**	**62.20 ± 0.70**
**∑ Polyunsat**			**16.55 ± 0.63**	**16.65 ± 0.99**
**∑ Plas**			**2.09 ± 0.32**	**1.85 ± 0.43**

Values (nanomole percent by weight composition) represent means ± standard errors. Means in the same row accompanied by asterisks are significantly different between treatments at LSD = 0.05, *n* = 12 per treatment group. D represents diacyl species and P represents plasmalogens species, p denotes a sn-1 vinyl ether (plasmalogen) linkage. Monounsat, monounsaturated fatty acids; PBS, phosphate buffered saline solution; plas, plasmalogens; polyunsat, polyunsaturated fatty acids; PPA, propionic acid. Phosphatidylcholine molecular species were identified using a precursor ion scan of *m/z* 184 in ESI positive mode.

**Table 4:** Changes (percent composition) in rat plasma phosphatidylcholine molecular species following intraventricular infusion with propionic acid (PPA) and phosphate buffered saline (PBS)

**Molecular weight**	**Molecular species**	**Plasmalogen or diacyl species**	**PBS**	**PPA**
706	D30:0	16:0/14:0	0.79 ± 0.42	0.59 ± 0.18
734	D32:0	16:0/16:0	1.39 ± 0.57	2.08 ± 0.14
718	P32:1	p16:0/16:1	0.52 ± 0.39	0.40 ± 0.053
762	D34:0	16:0/18:0	2.90 ± 0.34	3.80 ± 0.32*
760	D34:1	16:0/18:1	9.03 ± 0.73	9.98 ± 1.81
746	P34:1	p16:0/18:1	0.61 ± 0.28	0.52 ± 0.05
758	D34:2	16:0/18:2	20.13 ± 0.23	17.61 ± 0.16*
788	D36:1	18:0/18:1	8.11 ± 0.40	9.87 ± 0.38*
774	P36:1	p18:0/18:1	0.76 ± 0.24	6.24 ± 1.96*
782	D36:4	16:0/20:4	13.23 ± 0.42	11.41 ± 0.22*
814	D38:2	18:1/20:1	4.14 ± 0.24	2.97 ± 0.45*
812	D38:3	18:0/20:3	9.82 ± 2.05	8.70 ± 0.64
810	D38:4	18:0/20:4	23.92 ± 0.41	21.45 ± 0.87*
806	D38:6	16:0/22:6	2.42 ± 0.64	2.60 ± 0.63
834	D40:6	18:0/22:6	2.23 ± 0.68	1.78 ± 0.58
**Total%**			**100**	**100**
**∑ Saturates**			**3.68 ± 0.58**	**4.39 ± 1.21**
**∑ Monounsat**			**18.52 ± 1.57**	**21.92 ± 2.25**
**∑ Polyunsat**			**75.89 ± 2.55**	66.52 ± 2.09*
**∑ Plas**			**1.89 ± 0.86**	7.15 ± 1.84*

Values (nanomole percent by weight composition) represent means ± standard errors. Means in the same row accompanied by asterisks are significantly different between treatments at LSD = 0.05, *n* = 12 per treatment group. D represents diacyl species and P represents plasmalogens species, p denotes a sn-1 vinyl ether (plasmalogen) linkage., Monounsat, monounsaturated fatty acids; PBS, phosphate buffered saline solution; plas, plasmalogens; polyunsat, polyunsaturated fatty acids; PPA, propionic acid. Phosphatidylcholine molecular species were identified using a precursor ion scan of *m/z* 184 in ESI positive mode. The lipid components in the table are arranged based on the molecular species composition.

**Table 5:** Changes (percent composition) in rat brain phosphatidylinositol molecular species following intraventricular infusion with propionic acid (PPA) and phosphate buffered saline (PBS)

**Molecular weight**	**Molecular species**	**Diacyl species**	**PBS**	**PPA**
835	D34:1	16:0/18:1	0.53 ± 0.05	0.54 ± 0.07
859	D36:3	18:1/18:2	1.01 ± 0.14	0.79 ± 0.01*
857	D36:4	16:0/20:4	7.71 ± 0.32	7.02 ± 0.45
887	D38:3	18:0/20:3	9.07 ± 0.69	9.71 ± 0.73
885	D38:4	18:0/20:4	74.17 ± 1.35	74.60 ± 1.27
883	D38:5	18:1/20:4	5.53 ± 0.73	5.44 ± 0.41
881	D38:6	16:0/22:6	0.80 ± 0.04	0.79 ± 0.04
913	D40:4	18:0/22:4	0.41 ± 0.01	0.17 ± 0.03*
909	D40:6	18:0/22:6	0.77 ± 0.10	0.94 ± 0.12
**Total%**			**100**	**100**
**∑ Monounsat**			**0.53 ± 0.05**	**0.54 ± 0.07**
**∑ Polyunsat**			**99.46 ± 0.05**	**99.46 ± 0.07**

Values (nanomole percent by weight composition) represent means ± standard errors. Means in the same row accompanied by asterisks are significantly different between treatments at LSD = 0.05, *n* = 12 per treatment group. D represents diacyl species. Monounsat, monounsaturated fatty acids; PBS, phosphate buffered saline solution, polyunsat, polyunsaturated fatty acids; PPA, propionic acid. Phosphatidylinositol molecular species were identified using a precursor ion scan of *m/z* 241 in ESI negative mode. The lipid components in the table are arranged based on the molecular species composition.

**Table 6:** Changes (percent composition) in rat plasma phosphatidylinositol molecular species following intraventricular infusion with propionic acid (PPA) and phosphate buffered saline (PBS)

**Molecular weight**	**Molecular species**	**Diacyl/species**	**PBS**	**PPA**
835	D34:1	16:0/18:1	6.71 ± 0.66	9.04 ± 0.39*
859	D36:3	18:1/18:2	6.16 ± 0.29	8.67 ± 0.97*
857	D36:4	16:0/20:4	8.33 ± 1.03	8.70 ± 0.85
887	D38:3	18:0/20:3	8.53 ± 0.63	7.72 ± 0.69
885	D38:4	18:0/20:4	44.00 ± 2.19	32.68 ± 3.6*
883	D38:5	18:1/20:4	6.67 ± 0.56	7.79 ± 0.28
881	D38:6	16:0/22:6	6.17 ± 0.04	7.89 ± 0.50*
913	D40:4	18:0/22:4	6.50 ± 0.60	8.47 ± 0.31*
909	D40:6	18:0/22:6	6.93 ± 0.54	9.04 ± 0.38*
**Total%**			**100**	**100**
**∑ Monounsat**			**6.70 ± 0.66**	**9.03 ± 1.03**
**∑ Polyunsat**			**93.29 ± 0.66**	**90.96 ± 1.03**

Values (nanomole percent by weight composition) represent means ± standard errors. Means in the same row accompanied by asterisks are significantly different between treatments at LSD = 0.05, *n* = 12 per treatment group. D represents diacyl species. Monounsat, monounsaturated fatty acids; PBS, phosphate buffered saline solution, polyunsat, polyunsaturated fatty acids; PPA, propionic acid. Phosphatidylinositol molecular species were identified using a precursor ion scan of *m/z* 241 in ESI negative mode. The lipid components in the table are arranged based on the molecular species composition.

**Table 7:** Changes (nanomole percent by weight composition) in rat brain phosphatidylserine molecular species following intraventricular infusion with propionic acid (PPA) and phosphate buffered saline (PBS)

**Molecular weight**	**Molecular species**	**Diacyl species**	**PBS**	**PPA**
760	D34:1	16:0/18:1	0.90 ± 0.10	1.10 ± 0.21
790	D36:0	18:0/18:0	1.59 ± 0.41	1.65 ± 0.11
788	D36:1	18:0/18:1	18.10 ± 0.53	21.62 ± 0.63*
786	D36:2	18:0/18:2	2.36 ± 0.34	2.55 ± 0.17
816	D38:1	18:0/20:1	1.00 ± 0.15	1.22 ± 0.19
810	D38:4	18:0/20:4	4.18 ± 0.07	4.46 ± 0.12
840	D40:3	18:0/22:3	1.19 ± 0.23	1.20 ± 0.17
838	D40:4	18:0/22:4	4.67 ± 0.17	3.63 ± 0.14*
836	D40:5	18:0/22:5	6.40 ± 0.30	6.91 ± 0.43
834	D40:6	18:0/22:6	58.39 ± 0.98	54.49 ± 1.05*
832	D40:7	18:1/22:6	1.22 ± 0.08	1.17 ± 0.12
**Total%**			**100**	**100**
**∑ Saturates**			**1.59 ± 0.15**	**1.65 ± 0.11**
**∑ Monounsat**			**19.99 ± 0.39**	23.93 ± 0.66*
**∑ Polyunsat**			**78.41 ± 0.58**	74.45 ± 0.55*

Values (nanomole percent by weight composition) represent means ± standard errors. Means in the same row accompanied by asterisks are significantly different between treatments at LSD = 0.05, *n* = 12 per treatment group. D represents diacyl species. Monounsat, monounsaturated fatty acids; PBS, phosphate buffered saline solution; polyunsat, polyunsaturated fatty acids; PPA, propionic acid. Phosphatidylserine molecular species were identified using neutral loss scan of 87 D in ESI negative mode. The lipid components in the table are arranged based on the molecular species composition.

**Table 8:** Changes (nanomole percent by weight composition) in rat plasma phosphatidylserine molecular species following intraventricular infusion with propionic acid (PPA) and phosphate buffered saline (PBS)

**Molecular weight**	**Molecular species**	**Diacyl species**	**PBS**	**PPA**
762	D34:1	16:0/18:1	6.55 ± 0.30	7.94 ± 0.09*
792	D36:0	18:0/18:0	8.12 ± 0.57	7.62 ± 0.24
790	D36:1	18:0/18:1	9.53 ± 0.74	12.13 ± 1.21*
788	D36:2	18:0/18:2	7.81 ± 0.06	8.84 ± 0.63*
818	D38:1	18:0/20:1	7.72 ± 0.24	6.94 ± 0.63
812	D38:4	18:0/20:4	11.55 ± 1.36	11.76 ± 1.65
842	D40:3	18:0/22:3	8.64 ± 0.53	7.37 ± 0.51*
840	D40:4	18:0/22:4	7.81 ± 0.08	7.50 ± 0.35
838	D40:5	18:0/22:5	7.61 ± 0.23	7.69 ± 0.06
836	D40:6	18:0/22:6	16.67 ± 1.34	13.68 ± 0.88*
834	D40:7	18:1/22:6	7.99 ± 0.37	8.53 ± 0.19
**Total%**			**100**	**100**
**∑ Saturates**			**8.12 ± 0.57**	**7.62 ± 0.24**
**∑ Monounsat**			**23.80 ± 1.76**	27.02 ± 1.99*
**∑ Polyunsat**			**68.06 ± 0.99**	65.34 ± 1.18*

Values (nanomole percent by weight composition) represent means ± standard errors. Means in the same row accompanied by asterisks are significantly different between treatments at LSD = 0.05, *n* = 12 per treatment group. D represents diacyl species. Monounsat, monounsaturated fatty acids; PBS, phosphate buffered saline solution; polyunsat, polyunsaturated fatty acids; PPA, propionic acid. Phosphatidylserine molecular species were identified using neutral loss scan of 185 D in ESI positive mode. The lipid components in the table are arranged based on the molecular species composition.

**Table 9:** Changes (percent composition) in rat brain phosphatidylethanolamine molecular species following intraventricular infusion with propionic acid (PPA) and phosphate buffered saline (PBS)

**Molecular weight**	**Molecular species**	**Plasmalogen or diacyl species**	**PBS**	**PPA**
700	P34:1	p16:0/18:1	5.85 ± 0.15	5.10 ± 0.14*
714	D34:2	16:0/18:2	3.37 ± 0.17	3.49 ± 0.15
730	P36:0	p18:0/18:0	3.42 ± 0.13	4.03 ± 0.38
744	D36:1	18:0/18:1	3.50 ± 0.46	3.32 ± 0.37
728	P36:1	p18:0/18:1	6.00 ± 0.09	6.82 ± 0.12*
742	D36:2	18:0/18:2	3.68 ± 0.14	3.59 ± 0.35
726	P36:2	p18:0/18:2	4.79 ± 0.30	7.16 ± 0.62*
724	P36:3	p18:1/18:2	3.38 ± 0.42	2.98 ± 0.27
722	P36:4	p16:0/20:4	4.32 ± 0.12	4.43 ± 0.14
766	D38:4	18:0/20:4	6.99 ± 0.71	7.33 ± 0.62
750	P38:4	p18:0/20:4	6.07 ± 0.13	7.70 ± 0.02*
762	D38:6	16:0/22:6	4.18 ± 0.16	3.45 ± 0.15*
746	P38:6	p16:0/22:6	5.14 ± 0.45	5.11 ± 0.80
796	D40:3	18:0/22:3	3.59 ± 0.26	3.52 ± 0.43
794	D40:4	20:0/20:4	3.51 ± 0.08	3.85 ± 0.42
778	P40:4	p18:0/22:4	3.46 ± 0.26	5.00 ± 0.10*
792	D40:5	18:0/22:5	4.30 ± 0.31	3.93 ± 0.41
776	P40:5	p18:0/22:5	3.91 ± 0.69	3.79 ± 0.60
790	D40:6	18:0/22:6	11.07 ± 1.06	8.59 ± 0.36*
774	P40:6	p18:0/22:6	9.47 ± 0.21	6.82 ± 0.86*
**Total%**			**100**	**100**
**∑ Saturates**			**3.42 ± 0.12**	**4.02 ± 0.38**
**∑ Monounsat**			**15.32 ± 0.65**	**15.23 ± 0.92**
**∑ Polyunsat**			**81.23 ± 0.69**	**80.73 ± 0.55**
**∑ Plas**			**55.82 ± 1.63**	**58.93 ± 1.40**

Values (nanomole percent by weight composition) represent means ± standard errors. Means in the same row accompanied by asterisks are significantly different between treatments at LSD = 0.05, *n* = 12 per treatment group. D represents diacyl species and P represents plasmalogens species, p denotes a sn-1 vinyl ether (plasmalogen) linkage. Monounsat, monounsaturated fatty acids; PBS, phosphate buffered saline solution; plas, plasmalogens; polyunsat, polyunsaturated fatty acids; PPA, propionic acid. Phosphatidylethanolamine molecular species were identified using a precursor ion scan of *m/z* 196 in ESI negative mode. The lipid components in the table are arranged based on the molecular species composition.

**Table 10:** Changes (nanomole percent by weight composition) in rat plasma phosphatidylethanolamine molecular species following intraventricular infusion with propionic acid (PPA) and phosphate buffered saline (PBS)

**Molecular weight**	**Molecular species**	**Plasmalogen or diacyl species**	**PBS**	**PPA**
702	P34:1	p16:0/18:1	4.59 ± 0.43	3.51 ± 0.53
716	D34:2	16:0/18:2	4.42 ± 0.13	4.41 ± 0.66
732	P36:0	p18:0/18:0	4.39 ± 0.19	3.11 ± 0.41*
746	D36:1	18:0/18:1	4.55 ± 0.44	3.38 ± 0.49*
730	P36:1	p18:0/18:1	5.76 ± 0.56	3.57 ± 0.50 *
744	D36:2	18:0/18:2	5.20 ± 0.58	4.49 ± 0.55
728	P36:2	p18:0/18:2	3.10 ± 1.36	3.19 ± 0.26
726	P36:3	p18:1/18:2	4.28 ± 0.19	3.85 ± 0.36
724	P36:4	p16:0/20:4	4.47 ± 0.42	3.41 ± 0.46*
768	D38:4	18:0/20:4	10.82 ± 0.71	23.60 ± 4.93*
752	P38:4	p18:0/20:4	4.67 ± 0.06	3.70 ± 0.45*
764	D38:6	16:0/22:6	5.35 ± 0.21	7.02 ± 0.56*
748	P38:6	p16:0/22:6	4.87 ± 0.38	3.71 ± 0.64
798	D40:3	18:0/22:3	4.27 ± 0.34	3.92 ± 0.68
796	D40:4	20:0/20:4	4.78 ± 0.29	3.67 ± 0.62
780	P40:4	p18:0/22:4	4.06 ± 0.17	3.41 ± 0.34
794	D40:5	18:0/22:5	4.97 ± 0.35	4.07 ± 0.32
778	P40:5	p18:0/22:5	4.67 ± 0.27	3.76 ± 0.83
792	D40:6	18:0/22:6	5.89 ± 0.62	7.03 ± 1.63
776	P40:6	p18:0/22:6	4.80 ± 0.33	3.54 ± 0.69*
**Total%**			**100**	**100**
**∑ Saturates**			**4.39 ± 0.19**	3.11 ± 0.41*
**∑ Monounsat**			**14.92 ± 0.39**	10.46 ± 1.46*
**∑ Polyunsat**			**80.68 ± 0.43**	86.41 ± 1.81*
**∑ Plas**			**49.69 ± 0.96**	38.37 ± 3.81*

Values (nanomole percent by weight composition) represent means ± standard errors. Means in the same row accompanied by asterisks are significantly different between treatments at LSD = 0.05, *n* = 12 per treatment group. D represents diacyl species and P represents plasmalogens species, p denotes a sn-1 vinyl ether (plasmalogen) linkage. Monounsat, monounsaturated fatty acids; PBS, phosphate buffered saline solution, plas, plasmalogens; polyunsat, polyunsaturated fatty acids; PPA, propionic acid. Phosphatidylethanolamine molecular species were identified using neutral loss scan of 141 D in ESI positive mode. The lipid components in the table are arranged based on the molecular species composition.

### SM molecular species

Sphingomyelin molecular species containing saturated base (C18:0) and fatty acids predominate in brain samples (Table 1), while species with monounsaturated base (C18:1) and polyunsaturated fatty acid predominate in plasma samples (Table 2).

Propionic acid treatment resulted in alterations in the proportions of both brain (*F* (27, 56) = 10.79, *P* <0.001) and plasma ( *F* (27, 56) = 32.64, *P* <0.001) SM molecular species (Tables 1 and 2). In both brain (Table 1) and plasma (Table 2) an overall increase (*P* <0.001) was observed in the relative level of saturated species, in addition to a reduction in monounsaturated species. Polyunsaturated species were unchanged in brain, but increased in plasma following PPA infusions. Increased proportions ( *P* <0.001) of 36:0 (18:0/18:0) in brain (Table 1) and 38:0 (18:0/20:0) in plasma (Table 2) accounted for the increase in saturated SM species. In brain, the proportions of 38:0 (18:0/20:0) and 40:0 (18:0/22:0) were decreased, but this had no overall significant effect on the total brain proportions of saturated SM. Conversely, 38:1 (18:1/20:0) in brain (Table 1) and both 34:1 (18:1/16:0) and 38:1 (18:1/20:0) in plasma (Table 2) accounted for the reduction in monounsaturated SM species, while 42:3 (18:1/24:2) in plasma accounted for the increased polyunsaturated species following PPA infusions (Table 2).

### PC molecular species

Diacyl and plasmalogen PC molecular species were present in both brain (Table 3) and plasma (Table 4). However, only the diacyl species were altered (*F* (59, 120) = 1042.12, *P* <0.001) in brain following PPA treatments, while both the diacyl and plasmalogen species were altered ( *F* (59, 120) = 873.32, *P* <0.001) in plasma. In brain, the proportions of monounsaturated 34:1 (16:0/18:1) and polyunsaturated diacyl 34:2 (16:0/18:2) and 36:4 (16:0/20:4) species were increased; while a reduction was observed in the monounsaturated diacyl 36:1 (18:0/18:1) species following PPA treatment (Table 3). No overall change was observed in the relative proportions of saturated, unsaturated, and polyunsaturated PC species. Plasma samples on the other hand revealed a reduction in the level of diacyl polyunsaturated PC species (34:2 (16:0/18:2), 36:4 (16:0/20:4), 38:2 (18:1/20:1), 38:4 (18:0/20:4)), and an in increase in the proportions of plasmalogen species (36:1 (18:0/18:1)). Although plasma proportions of diacyl saturated 34:0 (16:0/18:0) and monounsaturated 36:1 (18:0/18:1) molecular species increased following PPA infusions, this had no effect on the overall level of total saturated and monounsaturated PC species (Table 4).

### PI molecular species

Propionic acid treatment altered the proportions of several plasma (*F* (35, 72) = 75.31, *P* <0.001) PI molecular species, while the proportions of only two brain ( *F* (35, 72) = 2017.70, *P* <0.001) PI molecular species were affected (slightly reduced) (Table 5 and 6). Reduced proportions of polyunsaturated diacyl 36:3 (18:1/18:2) and 40:4 (18:0/22:4) species were observed in brain samples following PPA treatment (Table 5). The alterations were more varied with plasma PI molecular species. The proportions of plasma diacyl 34:1 (16:0/18:1), 36:3 (18:1/18:2), 38:6 (16:0/22:6), 40:4 (18:0/22:4), and 40:6 (18:0/22:6) increased, while the proportions of the diacyl 38:4 (18:0/20:4) species decreased following PPA treatment (Table 6). These changes indicate a compositional shift occurred particularly in plasma PI molecular species following PPA infusions.

### PS molecular species

Propionic acid infusions altered the proportions of both mono and polyunsaturated PS species in brain (Table 7) and plasma (Table 8). In both brain (*F* (21, 44) = 305.49, *P* <0.001) and plasma ( *F* (21, 44) = 12.14, *P* <0.001), an overall increase was observed in the proportion of monounsaturated species, while a reduction was observed in polyunsaturated species. An increase in brain 36:1 (18:0/18:1) (Table 7) and plasma (Table 8) 34:1 (16:0/18:1) and 36:1 (18:0/18:1) accounted for the increase in monounsaturated PS species. Conversely, a reduction in brain proportions of 40:4 (18:0/22:4) and 40:6 (18:0/22:6) and plasma proportions of 40:3 (18:0/22:3) and 40:6 (18:0/22:6) accounted for the reduced polyunsaturated PS species following PPA infusions.

### PE molecular species

Compositional changes in brain diacyl and plasmalogen PE species occurred following PPA infusions (Table 9). Propionic acid treatment reduced (*F* (79, 160) = 8.79, *P* <0.001) *P* <0.001) brain relative proportions of plasmalogen 40:6 (p18:0/22:6), diacyl polyunsaturated 38:6 (16:0/22:6), and 40:6 (18:0/22:6) species, but increased the proportions of plasmalogen 36:1 (p18:0/18:1), 36:2 (p18:0/18:2), 38:4 (p18:0/20:4), and 40:4 (p18:0/22:4) molecular species. In plasma, both the composition and overall relative proportions of PE diacyl and plasmalogen species were altered following PPA infusions (Table 10). A reduction (*F* (79, 160) = 11.61, *P* <0.001) was observed in diacyl 36:1 (18:0/18:1), plasmalogen 36:0 (p18:0/18:0), 36:1 (p18:0/18:1), 36:4 (p16:0/20:4), 38:4 (p18:0/20:4), and 40:6 (p18:0/22:6) molecular species. In contrast, the proportions of polyunsaturated diacyl species (38:4 (18:0/20:4) and 38:6 (16:0/22:6)) increased with PPA infusions.

## Discussion

### Behavioral assessment

Locomotor activity as expressed by horizontal, vertical, and repetitive measures across infusion days was used to assess abnormal animal behavior. Assessment of locomotor activity indicates that PPA infusions induced hyperactivity and stereotypy in rodents consistent with the findings from previous studies by our group [7-12]. These behaviors bear some resemblance to the hyperactive and repetitive behaviors which are recognized as core symptoms of ASD [41]. PPA and other short chain fatty acids are known to increase intracellular neuronal and glial acidification and calcium proportions, thereby producing widespread effects on neurotransmitter release including glutamate, dopamine, norepinepherine, and serotonin, each of which play a role in elicitation of locomotor activity, as seen in the present study [42-44]. In addition, PPA has been shown to increase glutamatergic transmission, leading to excitability in brain regions linked to locomotor activity [45,46]. Collectively, these observations are consistent with an emerging theory of ASD as a disruption of excitatory/inhibitory neuronal activity [47]. The fact that PPA infusions consistently induce elevations in locomotor activity in rodents indicates that this model may be a useful tool for studying the neurological mechanisms involved in the behavioral disturbances seen in ASD.

### Alterations in phospholipid molecular species

The phospholipid profiles observed in control animals are consistent with those reported in the literature for rat brain ([48,49]with the exception of SM molecular species. Typically, molecular species with sphingosine base (C18:1) make up the largest proportion of the SM profile in mammals. In this study, molecular species with sphinganine bases (C18:0) accounted for the largest proportion of rat brain SM profile. At this time, we have no suitable/conclusive explanation for this observed variation in brain SM profile following careful perusal of the data to ensure mass spectral interpretation, data normalization, and calculations are correct to the best of our knowledge. However, a similar SM profile with saturated base predominating has been reported previously in human retina [40].

Phospholipids are the major structural components of neuronal and other cellular membranes, and include PC, PE, PS, PI, and SM [50,51]. All of these phospholipid classes were observed to have altered molecular species distribution following PPA infusion. It has been suggested that common neurodevelopmental disorders such as ASD could be associated with functional deficiencies or imbalances in fatty acid synthesis/metabolism [5,27,29,36]. Most of these former studies evaluated the fatty acid composition following hydrolysis of either total or individual phospholipid classes. Alterations in specific phospholipid molecular species could not only contribute more specific clinical criteria, but could also provide a basis for mechanistic interpretations. However, to date, only one study [27] has evaluated the intact phospholipid molecular species in the blood of ASD patients. Further, these authors only analyzed PE phospholipid molecular species. In the present study, we evaluated SM, PC, PE, PS, and PI molecular species following infusion with PPA and the induction of abnormal (ASD-like) behaviors. Alterations were observed in 21 brain and 30 blood phospholipid molecular species. Pastural *et al.*[27] observed elevations in the relative proportions of plasmalogen PE, saturated, and polyunsaturated PE molecular species in the plasma of ASD patients. In our plasma analyses we also observed elevations in the relative proportions of polyunsaturated PE species, but the relative reduction in plasmalogen PE was in contrast with their findings. In addition, plasma from PPA-infused rats demonstrated elevations in the relative proportions of some saturated SM and PC molecular species, diacyl and plasmalogen monounsaturated PC species, PS monounsaturated species, and some polyunsaturated PI molecular species (36:3, 40:4, and 40:6). Elevations were also observed in the proportions of some brain PC polyunsaturated molecular species, PS monounsaturated, and PE plasmalogen species in the present study.

Analysis of SM, PC, PI, PE, PS, and PC phospholipid classes were conducted with both brain and blood samples. In many cases, the same phospholipid molecular species were altered in both blood and brain, but the direction and the relative proportions of these alterations were not consistent between both sample types. Much of the published work demonstrating alterations in lipid metabolism in autism has been done using plasma obtained from ASD patients [5,27,32,36]. The findings from these studies include elevations in saturated fatty acids [27,29,30], accompanied by a decline in plasmalogens [5,29,30], mono and polyunsaturated (ω3 + ω6) fatty acids [5,29,30,32,36,52]. Conversely, others have reported elevations in the proportions of mono and poly unsaturated fatty acids [5,27,30,53]. However, the origin of these altered fatty acids and plasmalogens are unknown, because the structures of the phospholipids were destroyed by hydrolysis during sample analysis. This paper is the most comprehensive study to date which examines intact PL molecular species alteration in relation to autism. Currently, there is no clear uniform mechanism governing the etiology and early detection of ASD, and no accepted biomarkers are available. Although this study is descriptive in nature, it provides considerably more information than is currently available, and as such provides a foundation for defining which intact phospholipid molecular species can be altered in relation to ASD. This could provide a framework for future studies to elucidate the mechanisms associated with the observed lipid alterations and their relationship to behavioral changes in ASD.

The use of an animal model allowed comparison of brain and plasma PL during the period of PPA-induced ASD-like behaviors. The observations that small amounts (1.04 micromole/infusion) of PPA into brain can influence plasma lipid composition are considered intriguing. However, the plasma alterations noted did not correlate directly with those in brain. It could be that this difference merely reflects the nature of the PPA rodent model where treatment is limited to 8 days. Furthermore, ASD likely involves genetic, metabolic, and environmental factors which could result in systemic as well as CNS effects.

It is evident from the data presented in this study that PPA infusion produced small but significant alterations in the composition of brain and plasma phospholipid species. These alterations occurred independent of diet. The most notable alterations were observed in the composition of brain SM, diacyl mono and polyunsaturated PC, PI, PS, PE, and plasmalogen PC and PE molecular species. These observations are considered interesting because alterations in brain lipid composition, particularly during development can potentially have serious consequences on CNS function.

### Potential physiological consequences of altered phospholipid molecular species and their relation to ASD

#### *Lipid mediated signaling and neuroinflammation in ASD*

The pathological consequences of disturbances in phospholipid metabolism could include alterations in signal transduction involving the generation of second messengers derived from docosahexaenoic (C22:6n3) and arachidonic (C20:4n6) acids [54]. The observation that PPA infusion increased the proportions of brain PI and PC molecular species containing arachidonic acid and decreased the proportions of PS and PE molecular species with docosahexaenoic acid, suggests that PPA could influence the innate neuroinflammatory process observed in autism. Metabolism of arachidonic and docosahexaenoic acids released from the sn-2 position of the glycerol moiety by phospholipase A2 results in the formation of eicosanoids and docosanoids, respectively [54]. Both eicosanoids and docosanoids are potent modulators of the inflammatory response system. Eicosanoids are inflammatory mediators that induce the formation of proinflammatory cytokines such as tumor necrosis factor (TNF), interleukin 1 (IL-1), and interleukin 6 (IL-6). Elevated levels of TNF, IL-1, and IL-6 have been reported in plasma of ASD patients [28]. Docosanoids on the other hand are antiflammatory and include protectins and resolvins that have known neuroprotective effects [54]. There is now emerging evidence that autism may be accompanied by abnormalities in the inflammatory response system [28], and that this abnormality may be related to the increases in oxidative stress [55-57], innate neuroinflammation [58], and altered lipid profiles [5,27,30,32,53] reported in ASD.

The increased accumulation of brain molecular species with the eicosanoids precursor (arachidonic acid), and the reduced proportions of the molecular species containing the docosanoids precursor (docosahexaenoic acid) observed in this study are interesting, considering the increased innate neuroinflammation (reactive astrogliosis and activated microglia) previously observed with this model [7] and reported for the brain of ASD patients at autopsy [58].

Several of the studies analyzing the hydrolyzed fatty acid components obtained from the blood of ASD patients also report alterations in the proportions of arachidonic and docosahexaenoic acids [27,29,32,36]. This could indicate aberrations in fatty acid elongation and desaturation may occur in the etiology of ASD. Arachidonic and docosahexaenoic acids are cleaved from the sn-2 position of the glycerol moiety by phospholipase A2. Genetic sites linked to autism on chromosome 8q22 are in the proximity of the gene (8q24) for secretory phospholipase A2 [36]. Collectively, these findings suggest possible involvements of arachidonate, docosahexaenoate, and phospholipase A2 in the signal cascade associated with the innate neuroinflammation observed in ASD.

### Membrane fluidity and stability

Biological membranes are predominantly bilayers in which the inner and outer leaflets have different phospholipid compositions. In contrast, blood plasma phospholipids are present as monolayers, sourrounding lipoproteins, and the effect of fluidity on lipoprotein function is not well understood. Here we will refer to both cellular bilayers and blood lipoprotein monolayers collectively as ‘membranes’. Phospholipid molecular species distribution influences bilayer physical properties such as fluidity and this affects membrane protein function. Monolayer molecular species composition also affects physical properties. Organisms can adjust the order or fluidity of their cellular membranes in response to changes in their physiological environment by altering their lipid composition [50]. Alterations in brain and blood phospholipid composition consistent with an adjustment in the order or fluidity of the membrane in these tissues in response to PPA infusions were observed in this study. This adjustment was reflected by changes in the relative proportions of unsaturated, diacyl, and/or plasmalogen species. Alterations in desaturation can have profound changes on membrane fluidity because increased carbon-carbon double bonds make unsaturated fatty acids more mobile, flexible, and fluid [50]. Changes in the relative proportions and the composition of diacyl and plasmalogen forms of phospholipids can also impact the fluidity of the membrane. For example, plasmalogen species facilitate membrane fusion six times faster than diacyl species [59].

Phosphatidylcholine, which has a large polar head group does not pack closely in membrane bilayers and tends to be more fluid compared to PE, which has a small head group and packs more closely in membranes, making them less fluid at physiological temperatures. The PC/PE balance in cell membranes is thought to regulate membrane fluidity and stabilize the membrane [50,60]. In both brain and blood membranes, PPA infusions alter the PC and PE composition (both diacyl and plasmalogen forms) possibly disrupting this balance. Alterations in brain membrane lipid composition affecting fluidity have been found to be associated with a number of behavioral abnormalities, as well as neurological and psychiatric disorders in both adults and children [61]. Alterations in membrane fluidity affect membrane properties, which in turn can affect the functions of integral membrane proteins, ion channels and the permeability of solutes across the membrane [54]. It is unclear at this preliminary stage whether or not any of these processes are affected by PPA infusion in this model, or if they are related to the etiology of ASD. These are the subjects of further studies in our laboratory.

### Lipid oxidation/peroxisomal function

Plasmalogens are vinyl ether lipids found in PE, PC and PS. Several studies have reported increases [27] or declines [5,29,30] in plasmalogens obtain from the bloods of ASD patients. Typically plasmalogens have docosahexaenoic or arachidonic acids esterified in the sn-2 position of the glycerol moiety, and are essential for normal brain development and functions. Reduced plasmalogens and docosahexaenoic acid levels are characteristic of peroxisomal-associated neurological disorders such as infantile Refsum disease, adrenoleukodystrophy, adrenomyeloneuropathy, and Zellweger’s syndrome [5,27,59]. The only study we know that analyzed the intact PE molecular species of blood obtained from ASD patients observed an overall increase in PE plasmalogens species, and this increase was accompanied by an increase in docosahexaenoic acid containing molecular species [27]. In our rat model, we observed an overall reduction in the relative proportions of PE plasmalogens in plasma, inclusive of species with docosahexaenoic acid at the sn-2 position of the glycerol moiety. However, consistent with [27], elevated proportions of PE polyunsaturated diacyl species were observed in plasma, inclusive of docosahexaenoate containing species.

In brain, a reduction in the relative proportions of diacyl and plasmalogen PE molecular species containing docosahexaenoic acid was observed in this study following PPA infusion. This was accompanied by a relative increase in several other PE plasmalogen molecular species lacking docosahexaenoic acid in the sn-2 position of the glycerol moiety. Plasmalogens act as a reservoir for docosahexaenoic acid, and both compounds have synthetic steps that occur in the peroxisome, providing a biochemical link [5]. Peroxisomal disorders are characterized by abnormal peroxisomal biogenesis associated with altered functionality of the two rate limiting enzymes in plasmalogen synthesis, acyl-coenzyme A (CoA): dihydroxyacetonephosphate acyltransferase and alkyldihydoxyacetonephosphate synthase [36,59]. It appears from the findings presented in this study and those from previous reports [5,27,29,30,52] that aberrations in plasmalogen metabolism may occur in ASD, implying that peroxisomal dysfunction could be involved.

Plasmalogens are considered endogenous antioxidants, because their vinyl ether bonds are efficient neutralizers of reactive oxygen species, which damage the polyunsaturated fatty acids present in the sn-2 position of plasmalogen phospholipids [62]. The alterations observed in plasmalogen molecular species in relation to the alterations in brain and blood polyunsaturated PE and PC molecular species, may be a response to increase oxidation following PPA infusions. This is very interesting because oxidative damage of lipids has been suggested to play a part in the pathogenesis of many neurological diseases including autism [26,55]. In addition, increased oxidative stress, and decreased antioxidant capacity have been previously reported in this rodent model [7,8], and also found to be present in ASD patients [55-57]. Interestingly, oxidative damage has been shown to uncouple the gap junctions in astrocytes [63,64]. Arachidonic and docosahexaenoic acids which are very susceptible to oxidative damage have been shown to modulate the coupling capacity of gap junctions [65-67]. In this study, PPA infusion led to alterations in the proportions of brain arachidonic and docosahexaenoic acids containing molecular species.

Collectively, the findings presented in this study, along with those observed in the blood of ASD patients; indicate that alterations in peroxisomal associated lipid metabolism and increased oxidative stress, possibly predisposing polyunsaturated fatty acids to oxidative damage may be associated with the pathogenesis of ASD.

## Conclusion

Infusions with PPA-induced abnormal (ASD-like) behaviors in rodents consistent with previous studies. This induction in abnormal behaviors was accompanied by alterations in several brain and blood SM, PC, PS, PE, and PI molecular species. Alterations in lipid composition are known to affect membrane fluidity, peroxisomal functions, gap junction coupling capacity, and signaling during neuroinflammation, which may be associated with the pathogenesis of ASD, at least in a subset of patients. The mechanisms governing these findings and their potential relevance to the pathophysiology of ASD, particularly during development, is the subject of further studies in our laboratory. Finally, these PPA-induced alterations of brain and plasma phospholipid molecular species provide further validation of this rodent model as a useful tool to link the disparate behavioral, central nervous system, and systemic findings with a plausible environmental factor in ASD.

## Abbreviations

ASD: Autism spectrum disorder; BUT: Butyric acid; CNS: Central nervous system; ESI: MS electrospray ionization mass spectrometry; ICV: Intracerebroventricular; PBS: Phosphate buffered saline; PC: Phosphatidylcholine; PE: Phosphatidylethanolamine; PI: Phosphatidylinositol; PPA: Propionic acid; PS: Phosphatidylserine; PUFA: Polyunsaturated fatty acids; SM: Sphingomyelin.

## Competing interests

The authors declare no conflict of interest.

## Authors’ contributions

RHT designed and conducted the experiments for the lipid component of the study and wrote the manuscript. MMM designed and conducted the experiments for the behavioral component of the study and assisted in editing the manuscript. JRM assisted with lipid and data analysis and editing the manuscript. LT assisted with surgery and editing the manuscript. FP co-supervised the study, assisted with interpretations and editing the manuscript. SL assisted with electrospray analysis and editing the manuscript. DFM supervised the study and assisted with editing the manuscript. All authors discussed the results/ implications, commented on the manuscript at all stages; read and approved the final manuscript.
